# Supplementary material for: Complications of Central Venous Access Devices Used in Palliative Care Settings for Terminally Ill Cancer Patients: A Systematic Review and Meta-Analysis
Source: Cancers (Basel). 2023 Sep 25;15(19):4712. doi: 10.3390/cancers15194712 (PMC10571956; doi:10.3390/cancers15194712)
Supplement: Supplementary file 1 [file cancers-15-04712-s001.zip › cancers-2558918-supplementary.pdf]

Supplementary Materials

Supplementary Method

Literature search strategy and criteria

(((central venous catheter[title/abstract] OR central venous access[title/abstract] OR central line[title/abstract] OR Hickman[title/abstract] OR Hickman catheter[title/abstract] OR PORT[title/abstract] OR Port-a-cath[title/abstract] OR PORT catheter[title/abstract] OR PICC[title/abstract] OR peripherally inserted central catheter[title/abstract] AND cancer[title/abstract] OR cancers[title/abstract] AND palliative care[title/abstract] OR terminally ill[title/abstract] OR critically ill[title/abstract]))) AND ("0001/01/01"[Date - Publication] : "2023/04/30"[Date - Publication])

PICOS framework for systematic review and meta-analysis

P (Patient or population) – Cancer patients receiving palliative care or best supportive care

I (Intervention) – Insertion of central venous access devices

C (Comparison) – Comparison of various types of central venous access devices including peripherally inserted catheter, central line or totally imported port

O (Outcome) – Complications related to insertion of central venous access devices including overall complication rate, rate of catheter-related bloodstream infection, and rate of thromboembolism

S (Study design) – Systematic review and meta-analysis

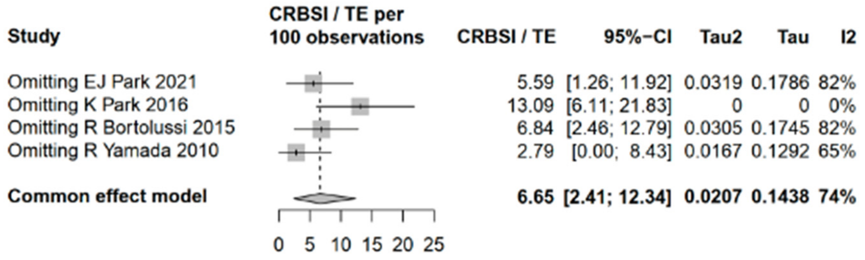

(a)

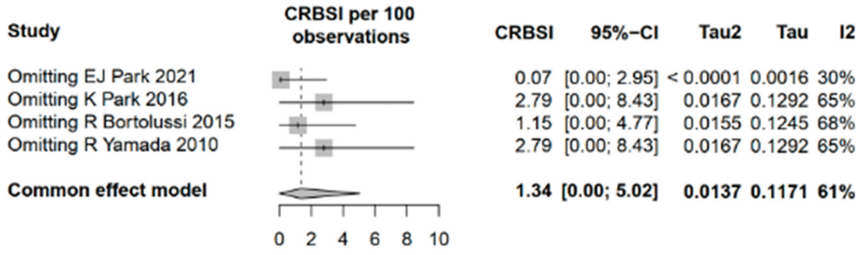

(b)

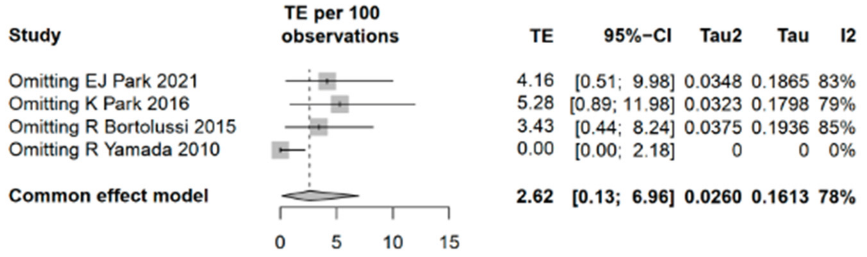

(c)

Supplementary Figure S1. Forest plot showing leave-one-out meta-analysis for PICC in (**a**) catheter-related bloodstream infection (CRBSI) and thromboembolism (TE), (**b**) CRBSI, and (**c**) TE.
